# Physical and sexual victimization of persons with severe mental illness seeking care in central and southwestern Uganda

**DOI:** 10.3389/fpubh.2023.1167076

**Published:** 2023-08-09

**Authors:** Richard Stephen Mpango, Wilber Ssembajjwe, Godfrey Zari Rukundo, Philip Amanyire, Carol Birungi, Allan Kalungi, Rwamahe Rutakumwa, Christine Tusiime, Kenneth D. Gadow, Vikram Patel, Moffat Nyirenda, Eugene Kinyanda

**Affiliations:** ^1^Mental Health Section, MRC/UVRI and LSHTM Uganda Research Unit, Entebbe, Uganda; ^2^Brown School, Washington University in St. Louis, St. Louis, MO, United States; ^3^Department of Mental Health, School of Health Sciences, Soroti University, Soroti, Uganda; ^4^Statistical Section, MRC/UVRI and LSHTM Uganda Research Unit, Entebbe, Uganda; ^5^Department of Psychiatry, Mbarara University of Science and Technology, Mbarara, Uganda; ^6^Department of Nursing, Mbarara University of Science and Technology, Mbarara, Uganda; ^7^Department of Psychiatry, College of Health Sciences, Makerere University, Kampala, Uganda; ^8^Butabika National Psychiatric Hospital, Kampala, Uganda; ^9^Department of Psychiatry, Stony Brook University, Stony Brook, NY, United States; ^10^Department of Global Health and Social Medicine, Harvard Medical School, Boston, MA, United States; ^11^Global Non-Communicable Diseases (NCD) Section, MRC/UVRI and LSHTM Uganda Research Unit, Entebbe, Uganda

**Keywords:** physical victimization, sexual victimization, mental illness, seeking care, Uganda

## Abstract

**Purpose:**

This study established the prevalence of physical and sexual victimization, associated factors and psychosocial consequences of victimization among 1,201 out-patients with severe mental illness at Butabika and Masaka hospitals in Uganda.

**Methods:**

Participants completed structured, standardized and locally translated instruments. Physical and sexual victimization was assessed using the modified adverse life events module of the European Para-suicide Interview Schedule. We used logistic regression to determine the association between victimization, the associated factors and psychosocial consequences.

**Results:**

The prevalence of physical abuse was 34.1% and that of sexual victimization was 21.9%. The age group of > = 50 years (aOR 1.02;95% CI 0.62–1.66; *p* = 0.048) was more likely to have suffered physical victimization, while living in a rural area was protective against physical (aOR 0.59; 95% CI 0.46–0.76; *p* = <0.001) and sexual (aOR 0.48, 95% CI 0.35–0.65; *p* < 0.001) victimization. High socioeconomic status (SES) (aOR 0.56; 95% CI 0.34–0.92; *p* = <0.001) was protective against physical victimization. Females were more likely to have been sexually victimized (aOR 3.38; 95% CI 2.47–4.64; *p* = <0.001), while being a Muslim (aOR 0.60; 95% CI 0.39–0.90; *p* = 0.045) was protective against sexual victimization. Risky sexual behavior was a negative outcome associated with physical (aOR 2.19; 95% CI 1.66–2.90; *p* = <0.001) and sexual (aOR 3.09; 95% CI 2.25–4.23; *p* < 0.001) victimization. Mental health stigma was a negative outcome associated with physical (aOR 1.03; 95% CI 1.01–1.05; *p* < 0.001) and sexual (aOR 1.03; 95% CI 1.01–1.05; *p* = 0.002) victimization. Poor adherence to oral anti-psychotic medications was a negative outcome associated with physical (aOR 1.51; 95% CI 1.13–2.00; *p* = 0.006) and sexual (aOR 1.39; 95% CI 0.99–1.94; *p* = 0.044) victimization.

**Conclusion:**

There is a high burden of physical and sexual victimization among people with SMI in central Uganda. There is need to put in place and evaluate complex interventions for improving detection and response to abusive experiences within mental health services. Public health practitioners, policymakers, and legislators should act to protect the health and rights of people with SMI in resource poor settings.

## Introduction

Much of the research on violence and severe mental illness (SMI) has focused on violence committed by individuals with mental illness with their victimization experience receiving little attention (1). This has contributed in a large measure to the stereotyping of persons with SMI as ‘violent and unpredictable’ helping to drive the stigma associated with the mental illness label (1). Contrary to this perception, emerging literature indicates that individuals with SMI are at significantly higher risk of violent victimization compared to the general population (2–5).

In a systematic review by Latalova et al. (4), the prevalence of violent victimization in the last 1 year was reported to range between 7.1 and 56% (4). Risk factors for both sexual and physical victimization have been reported to include: socio-demographic factors (male/female gender-depending on the study, homelessness, residence in a poor neighborhood); psychiatric illness factors (severity of psychiatric symptomatology, comorbid personality disorder); and psychosocial factors [engagement in criminal activity, stress (for men), history of victimization in childhood and adolescence and recent history of violence perpetration] (1, 3, 5–7).

Three theories suggested by Siegel (8) seem to apply to victimization of persons with SMI (8); the first victim perception theory suggests that some people may actually initiate the confrontation that eventually leads to their injury or death. For example, through pursuit of a relationship with the perpetrator or through personality traits such impulsivity that might render them abrasive or obnoxious. The second theory states that people may become crime victims because their lifestyle increases their exposure to criminal offenders, for example members of high risk groups such as the homeless and drug users. The third theory states that the more often victims visit dangerous places, the more likely they will be exposed to crime and violence for example if they reside in socially disorganized high-crime areas (8). Violent victimization of persons with SMI is of public health concern because of its known negative impact on the course and outcomes of mental disorder (9). Violent victimization of persons with SMI has been associated with increased severity of psychiatric symptoms, self-harm behavior, chronicity, increased risk of homelessness, reduced quality of life, impaired community functioning and greater caregiver financial burden (4, 10, 11). While considerable research has been undertaken to elucidate this problem in western countries, there is a paucity of research from developing country settings such as those in sub-Saharan Africa. In this paper we investigate the prevalence, risk factors and psychosocial consequences of physical and sexual victimization of persons with severe mental illness seeking care at two psychiatric facilities in central and south-western Uganda.

## Materials and methods

### Study design and site

This is a cross-sectional analysis from the longitudinal study entitled, ‘*HIV clinical trials preparedness studies among patients with severe mental illness in HIV endemic Uganda-the SMILE Study*’ (12, 13). Baseline recruitment included 1,201 individuals with severe mental illness (SMI) who were enrolled from the out-patient departments of Butabika National Referral Mental Hospital (urban central) and the Department of Psychiatry, Masaka Regional Referral Hospital (rural southwestern) Uganda between January–March 2018. Butabika National Referral Mental Hospital offers general and specialized mental health services both to in-patients and out-patients. Butabika National Referral Mental Hospital has a current psychiatric bed occupancy of 1,100 in-patients and sees about 30,000 psychiatric out-patients annually (personal communication from the Executive Director, Butabika National Psychiatric Referral Hospital, 23rd September 2022). Masaka Regional Referral Hospital offers all services expected of a regional referral health facility, including psychiatric services. The Psychiatric Department at Masaka Regional Referral Hospital has a 30-bed capacity in-patient service and an out-patient service. In the period between July 2018 to June 2019, the psychiatric department at Masaka Regional Referral Hospital attended to 8,260 out-patients (14, 15).

### Eligibility criteria

Serious Mental Illness (SMI) was operationalized as a condition whereby someone over the age of 18 years has (or had within the past year) one or more of the following diagnosable mental disorders: schizophrenia, bipolar affective disorder and recurrent major depressive disorder that caused serious functional impairment leading to at least one admission. The diagnosis was confirmed by a review of the clinical records by a psychiatrist (member of the research team). At the time of enrollment into the study, the study participant must have been in remission and attending the out-patient departments of either Butabika National Referral Mental Hospital or Masaka Regional Referral Hospital. Additional eligibility criteria included speaking either English or Luganda (the local language spoken in the study areas). Exclusion criteria were concurrent enrollment in another study, in need of immediate medical attention, and unable to understand the study assessment instruments for whatever reason.

### Recruitment and data collection

Participants were randomly selected from over 3,000 recovering mentally sick people attending out-patients’ departments (OPDs) at Butabika National Referral Mental Hospital and Masaka Regional Referral Hospital between January–March 2018 (study flow chart is below).

The trained research assistants (Psychiatric Clinical Officers and Psychiatric Nurses) gave potential participants information about the study before obtaining informed consent and assent to enroll into the study. Research assistants collected the data using structured, standardized, and locally translated assessment instruments (16–19). Participants with predetermined high risk criteria (as determined by the MINI criteria) or severe psychiatric symptomatology were referred to attending clinicians in the out-patient departments of the two participating hospitals.

### Measures

**Figure 1:**
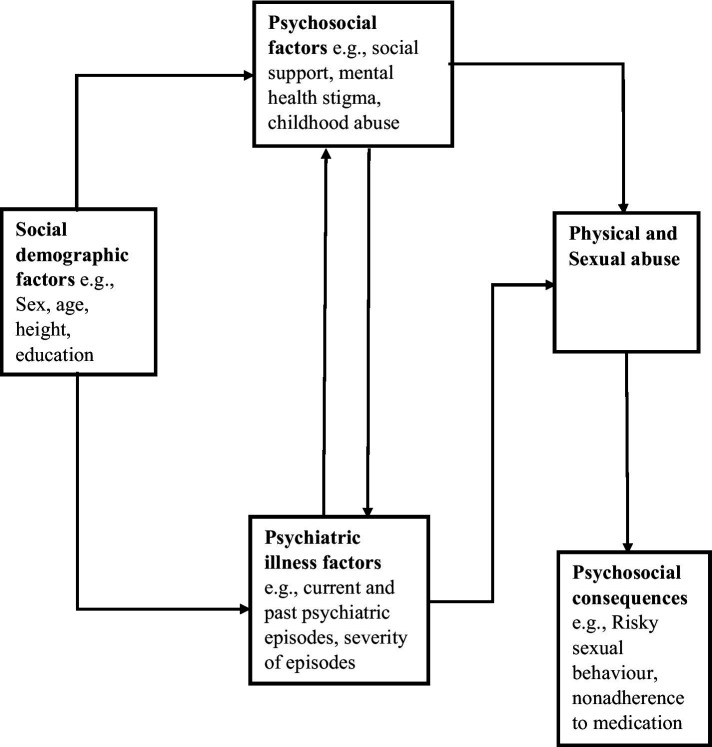
Conceptual framework for physical and sexual abuse among people with severe mental illness.

SMIs were established using the MINI International Neuropsychiatric Interview version 7.2. The variables reported in this paper were organized under the sub-headings based upon the conceptual framework developed by the ‘SMILE study team’ (Figure 1) (12, 13): (i) socio-demographic factors (study site, gender, age category, religion, socio-economic status, and marital status), (ii) psychosocial factors (social support, mental health stigma, childhood physical victimization, childhood sexual victimization, physical victimization in adulthood and sexual victimization in adulthood), (iii) psychiatric illness factors (family history of psychiatric illness, past depressive episode, past manic episode, past psychotic episode, lifetime suicide attempt), (iv) psychotropic drugs (Antiparkinsonian medication, mood stabilizers, 1st generation neuroleptics, 2nd generation neuroleptics, tri-cyclic anti-depressants, selective serotonin reuptake Inhibitors) and (v) maladaptive behaviors (alcohol use, use of tobacco, alcohol drinking problem, use of marijuana or use of khat).

### Statistical analysis

In this study, we had two sets of dependent variables; namely, adulthood physical and sexual victimization variables and clinical and behavioral outcome variables (risky sexual behavior, poor adherence to oral anti-psychotic medications). Frequencies of socio-demographic characteristics (study site, gender, age, socio-economic status, marital status, employment status, religion and education level) were described with frequencies and percentages for the categorical variables and median (IQR) for the continuous variables.

For each of the two outcome categories on adulthood victimization namely, physical and sexual victimization, three derived outcome variable were constructed from the relevant sections of the Uganda modified life events and histories module of the European Parasuicide Study Interview Schedule I (EPSIS I) (16, 20). The three derived outcome categories were: ‘*past adulthood physical or sexual victimization*’ (between 18 years of age to 12 months before interview date); ‘*recent adulthood physical or sexual victimization*’ (in the last 12 months before the study); and ‘*ever suffered physical or sexual victimization in adulthood*’ (between 18 year of age to interview date). The first two categories were derived from the relevant section of the life events and histories module of EPSIS I while the last category was a combination of the two former categories.

Prevalence of each of the three derived outcome variables for both physical and sexual victimization was calculated with 95% confidence intervals. To assess for factors associated with the derived outcome variables of ‘*ever suffered physical victimization in adulthood*’ (between 18 year of age to interview date) and ‘*ever suffered sexual victimization in adulthood*’ (between 18 year of age to the interview date) the following approach was employed.

To undertake multivariate analyses, the approach recommended by Victora et al. (21) was used based on the conceptual framework. Firstly, the association of socio-demographic factors was investigated through the use of a backward elimination logistic regression model, choosing the candidate variables based on prior knowledge and plausibility, and using a liberal *p*-value (15%) for removal, in order to ensure that all variables that could have a possible confounding effect on the ultimate risk factors were included, as recommended by Royston et al. (22). At second stage of model building, psychiatric illness factors and psychosocial factors were included into the model at a liberal value of *p* of 15%.

To investigate the association between clinical and behavioral outcome variables with physical and sexual victimization, the following approach was employed. For continuous outcomes ordinal logistic regression models were fitted while for the binary outcomes (risky sexual behavior, adherence to oral anti-psychotic medications), logistic regression models were fitted. At each of these model building stages, the likelihood ratio approach was used to determine the best fit for the final models. The following covariates were also controlled for as design variables: age, gender and study site. A two-sided *p* < 0.05 was considered statistically significant. STATA version 15.0 was used for all statistical analyses.

### Ethical consideration

The study obtained ethical approvals from the Uganda Virus Research Institute’s Research and Ethics Committee (GC/127/19/10/612) and the Uganda National Council of Science and Technology (HS 2337). Participants were given information about the study by trained study psychiatric nurses and informed consent and assent sought before enrolment into the study. Participants found to have a SMI were provided healthcare and supported at the out-patient departments (OPDs) of their respective hospitals.

## Results

### Characteristics of study participants

Out of the 1201 participants enrolled into the study, 54.5% were female and 58.4% lived in urban areas (Table 1). The average age of the participants was 36 years ±11.9, the majority (81.3%) were Christians and over 95% had attained at least primary education. Participants who were HIV seropositive were 7.2%, while those who were syphilis seropositive were 4.4% as indicated in Table 1.

**Table 1:** Baseline characteristics.

Factor	Level	*N* = 1,201 *n* (%)
Site	Butabika(urban)	701 (58.4%)
	Masaka(rural)	500 (41.6%)
Gender	Male	547 (45.5%)
	Female	654 (54.5%)
Age	Median(IQR)	36 (29,45)
Socio-economic status(grouped)	0–2	189 (15.7%)
	3–4	409 (34.1%)
	5–6	486 (40.5%)
	8-Jul	117 (9.7%)
Marital status	Currently married	384 (32.0%)
	Widowed	57 (4.7%)
	Separated/divorced	295 (24.6%)
	Single	464 (38.7%)
Employment status	Farmer/fisherman	304 (25.4%)
	Professional	139 (11.6%)
	Informal employment	206 (17.2%)
	Unemployed	549 (45.8%)
Religion	Christian	977 (81.3%)
	Muslim	212 (17.6%)
	Others	10 (0.83%)
Education level	No formal education	37 (3.1%)
	Primary	476 (39.6%)
	Secondary	460 (38.3%)
	Tertiary	227 (18.9%)
Primary diagnosis	Schizophrenia	314 (26.1%)
	Bipolar affective disorder	787 (65.5%)
	Major Depressive disorder	83 (6.9%)
Missed taking oral psychiatric medications in last 3 days	Yes	260 (21.7%)
	No	912 (75.9%)
HIV status	Positive	87 (7.2%)
Syphilis (VDRL)	Positive	53 (4.4%)

### Prevalence of physical and sexual victimization

Four hundred and nine (34.1%) participants met criteria for ‘ever suffered adulthood physical victimization’ with ‘recent adulthood physical victimization’ (in the last 12 months) reported by 13.6%, while ‘past physical victimization in adulthood’ (from 18 years up to 12 months before study) was reported by 31.1%. Two hundred and sixty-three (21.9%) participants met criteria for ‘ever suffered adulthood sexual victimization’ with ‘recent adulthood sexual victimization’ (in the last 12 months) reported by 8.6%, while ‘past sexual victimization in adulthood’ (from 18 years up to 12 months before study) was reported by 19.6% (Tables 2, 3).

**Table 2:** Prevalence of physical and sexual victimization.

	*n*	Prevalence	95%CI
Ever suffered adulthood Physical victimization (between 18 year of age to interview date)	409/1201	34.1%	(31.4–36.8%)
Past physical victimization in adulthood (*from 18 years up to 12 months before study*)	373/1200	31.1%	(28.5–33.8%)
*Recent adulthood physical victimization (in the last 12 months)*	163/1199	13.6%	(11.8–15.6%)
Ever suffered adulthood Sexual victimization (between 18 year of age to interview date)	263/1201	21.9%	(19.7–24.3%)
Past sexual victimization in adulthood (*from 18 years up to 12 months before study*)	237/1200	19.6%	(17.5–22.1%)
*Recent adulthood sexual victimization (in the last 12 months)*	103/1095	8.6%	(7.1–10.3%)

**Table 3:** A prevalence of sexual and physical victimization by severe mental illness diagnosis.

	Prevalence 95%CI		
	Schizophrenia	Bipolar affective disorder	Major depressive disorder
Sexual victimization	22.3 (18.0; 27.2)	21.6 (18.9; 24.6)	22.9 (15.0; 33.3)
Physical victimization	36.6 (31.4; 42.1)	33.8 (30.6; 37.2)	25.3 (17.0; 35.9)

### Socio-demographic and psychosocial factors associated with physical and sexual victimization

Age (> = 50) of the respondent was found to be associated with physical victimization (*p* = 0.048). Residing in rural residences (Masaka) was found to be protective against physical (aOR 0.59, 95% CI 0.46–0.76; *p* < 0.001) and sexual (aOR 0.48, 95% CI 0.35–0.65; *p* < 0.001) victimization. Socio-economic status was also found to be associated with physical victimization; participants with a higher socio-economic status were less likely to suffer physical victimization compared to those with lower socio-economic status.

Sex of the respondent was found to be associated with sexual victimization. Female respondents reported three times more likely to suffer sexual victimization compared to their male counterparts (aOR 3.38; 95% CI 2.47–4.64; *p* < 0.001). Religion (being Muslim) was protective by 40% against sexual victimization (*p* = 0.045) (Table 4). Childhood trauma was associated with both physical (aOR 1.04; 95% CI 1.03–1.05; *p* < 0.001) and sexual (aOR 1.05; 95% CI 1.04–1.06; *p* < 0.001) victimization. Social support was protective against both physical (aOR 0.95; 95% CI 0.94–0.97; *p* < 0.001) and sexual (aOR 0.96; 95% CI 0.94–0.98; *p* < 0.001) victimization.

**Table 4:** Socio-demographic and psychosocial factors associated with physical and sexual victimization.

Factor	Level	Physical victimization	*p*–value	Sexual victimization	*p*-value
		Adj. OR		Adj. OR	
Age	<25	1			
	25–34	1.55 (1.01,2.38)		–	
	35–49	1.46 (0.96,2.23)			
	> = 50	1.02 (0.62,1.66)	0.048		
Sex	Female	0.85 (0.67,1.08)		3.38 (2.47;4.64)	<0.001
	Male	1	0.188	1	
Urban/rural residence	Butabika	1		1	
	Masaka	0.59 (0.46,0.76)	<0.001	0.48 (0.35;0.65)	<0.001
Religion	Christian			1	
	Muslim	–		0.60 (0.39;0.90)	0.045
	Others			1.32 (0.27;6.51)	
Marital status	Currently	1			
	Widowed	0.96 (0.49,1.87)		–	
	Separated	1.48 (1.07,2.05)			
	Single	1.09 (0.80,1.49)	0.091		
Socio-economic status	2-January	1			
(number of items possessed)	4-March	0.68 (0.48,0.98)		-	
	6-May	0.43 (0.30,0.62)			
	8-July	0.56 (0.34,0.92)	<0.001		
Employed/unemployed	Farmer			1	
	Professional	–		1.10 (0.66;1.84)	0.079
	Informal			0.57 (0.34;0.97)	
	unemployed			1.01 (0.69;1.49)	
Childhood trauma	Per unit increase	1.04 (1.03,1.05)	<0.001	1.05 (1.04;1.06)	<0.001
Social support	Per unit increase	0.95 (0.94,0.97)	<0.001	0.96 (0.94;0.98)	<0.001
Primary diagnosis	Schizophrenia	1		1	
	Bipolar affective disorder	1.01 (0.76; 1.34)		1.09 (0.77; 1.53)	
	Major depressive diosrder	0.67 (0.38; 1.17)	0.301	1.05 (0.57; 1.93)	0.882

All analyses adjusted for study site, sex and age.

### Association between physical and sexual victimization with negative outcomes

Risky sexual behavior was associated with both physical (aOR 2.19; 95% CI 1.66–2.90; *p* < 0.001) and sexual (aOR 3.09; 95% CI 2.25–4.23; *p* < 0.001) victimization. Mental health stigma (Per unit increase) was associated with physical (aOR1.03; 95% CI 1.01–1.05; *p* < 0.001) and sexual (aOR 1.03; 95% CI 1.01–1.05; *p* = 0.002) victimization. Missed taking of oral psychiatric medications in last 3 days was associated with both physical (aOR1.50; 95% CI 1.12–2.00; *p* = 0.006) and sexual (aOR 1.39; 95% CI 1.00–1.93; *p* = 0.044) victimization. Poor adherence to oral anti-psychotic medications was associated with both physical (aOR 1.51; 95% CI 1.13–2.00; *p* = 0.006) and sexual (aOR 1.39; 95% CI 0.99–1.94; *p* = 0.044) victimization (Table 5).

**Table 5:** Association between physical and sexual victimization with negative outcomes.

Factor		Any physical victimization	*p*-value	Any sexual victimization	*p*-value
		Adj. OR		Adj. OR	
Behavioral outcomes					
Risky sexual behavior	Yes	2.19 (1.66; 2.90)	<0.001	3.09 (2.25;4.23)	<0.001
Mental health stigma	Per unit increase	1.03 (1.01,1.05)	<0.001	1.03 (1.01;1.05)	0.002
Clinical outcomes					
Missed taking oral psychiatric medications in last 3 days	Yes	1.50 (1.12; 2.00)	0.006	1.39 (1.00; 1.93)	0.044
Poor adherence to oral anti-psychotic medications	Yes	1.51 (1.13; 2.00)	0.006	1.39 (0.99;1.94)	0.044

All analyses adjusted for study site, sex and age.

## Discussion

This study demonstrates a high prevalence of physical and sexual victimization, associated factors and negative outcomes among patients with severe mental illness (SMI) attending out-patients’ departments (OPDs) at Butabika and Masaka hospitals in Uganda. The prevalence of physical victimization was 34.1%, while sexual victimization was 21.9%. Associated risk factors for both physical and sexual victimization were, living in a rural area, childhood trauma, and social support; Age group of > = 50 and high social economic status (SES) were associated with physical victimization, while female gender and religion (being a Muslim) were associated with sexual victimization. Negative outcomes associated with both physical and sexual victimization were risky sexual behaviors, mental health stigma, having missed to take oral psychiatric medications in last 3 days, and poor adherence to oral anti-psychotic medications.

### Prevalence of physical and sexual victimization

According to this study, the prevalence of physical victimization among people with SMI (34.1%) was similar to the rate of 31% established by Frueh et al. (23, 24) but much higher than the rate of 20.7% (in women) and 17.8% (in men) that was established by a recent review of 30 studies about victimization against people with SMI (25); the prevalence of physical victimization established by this study relates to the national prevalence of physical victimization among women (34%) and men (45%) in the general population in Uganda (26). Compared to people with SMI versus those without SMI, the odds of physical victimization happen to be elevated with a range of 2–22-fold higher odds (with a recent study reporting a pooled estimate of 5-fold relative odds for any physical victimization among people with SMI compared to those without SMI) (25); similarly, a previous systematic review by Hughes et al. (27) established a 4-fold risk for any physical victimization among people with disabilities compared to those without disabilities. This study established a prevalence of sexual victimization (21.9%) which was much higher than the established rate of 8% by Frueh et al. (23) and higher than 9.9% (in Women) and 3.1% (in men) established by a recent review of 30 studies that focused upon victimization among people with SMI (25); the prevalence of sexual victimization established by this study was much higher than the national prevalence of sexual victimization among women (5%) and men (2%) in the general population in Uganda (26). The prevalence of physical and sexual victimization established by this study relates to the prevalence of physical and sexual intimate partner violence (IPV) among women living with HIV (WLWH) (29%) (28), but higher than the national prevalence of both physical and sexual victimization among women (18%) and men (6%) in Uganda (26). Several mechanisms have been suggested to explain the high prevalence of physical and sexual victimization among people with SMI; living in socially deprived neighborhoods (with its social and economic conditions) fosters physical and sexual victimization among people with SMI (25, 29), but such deprived neighborhoods (with such social and economic conditions) also foster physical and sexual victimization among people without SMI (25, 29). Another mechanism suggests that some people with SMI (acutely ill patients) display disturbed or psychotic behavior, which may evoke hostile reactions and attempts at social control from others, often results into conflict and mutual victimization (30). Exposure to institutional victimization (i.e., coercive measures such as being taken down by police or psychiatric staff, being committed against own will, forced medication, seclusion, or restraint) within the mental health care system has been shown to lead to physical victimization (sometimes sexual victimization, especially when a ‘staff contact person’ is absent or treatment by staff happens to be dismissive and derogatory) (31–33). Similarly, homelessness among people with SMI is highly associated with increased physical and sexual victimization (25, 34, 35); possibly, the poor development of mental health facilities, poor staffing levels [with increased use of ‘informally trained low-level staff’ (‘local security-guards’) to offer clinical services, e.g., use of batons to manage aggressive/violent patients], poor ‘qualified staff-to-patient-ratios (especially during the night shifts)’, poor infrastructure of the mental health services, coupled with inadequate sensitization could partly be responsible for the increased prevalence of physical and sexual victimization among people with SMI in Uganda compared to other parts of the world.

### Socio-demographic and psychosocial factors associated with physical and sexual victimization

Result from this study indicate that the age category of > = 50 years was associated with physical victimization (*p* = 0.048), contrary to a previous study which established that victimization rates among adults with SMI decrease with age (4); increased physical victimization with age established by this study possibly relates to the increased illness severity (number of hospitalizations, number of symptoms) with increasing age, coupled with lack of meaningful social roles for the majority of people with SMI which makes them vulnerable to victimization (25). According to this study, age of the respondent was not associated with sexual victimization; findings from this study were similar to a previous study which established that people with severe mental illness experience victimization, regardless of their age (25), but contrary to findings from a study which established that younger age is associated with sexual victimization, while victimization rates appeared to decrease with age (4). This study established that female respondents were three times more likely to suffer sexual victimization compared to their male counterparts; findings from this study were similar to a previous study which established that the prevalence of sexual victimization was three times higher in women [9.9% (IQR ¼ 5.9–18.1%)] as compared to men [3.1% (IQR ¼ 2.5–6.7%)] (25); results from this study were in agreement with previous studies which suggest that women are more likely to be victims of sexual abuse (4, 25). Similarly, other studies from high-income countries suggest that women with SMI are at an increased risk of sexual victimization both within and outside intimate relationships (34, 36–38). Female patients are significantly more likely to be sexually victimized than male patients (4, 39), because being female is associated with an increased risk of victimization (4). In sub-Saharan Africa, access to mental health treatment is limited, thus women with inadequately treated psychiatric symptoms constitute ‘suitable targets’ for sexual victimization (34, 40). According to this study, residing in rural residences was found to be protective by 41% against physical victimization, which rhymes with a previous study which revealed that living in rural areas is significantly associated with lower risk of reporting severe mental illnesses and happens to be associated with better overall mental health (41); additionally, subjective safety for people with SMI is clearly worse in cities than in rural areas (42), while other related studies established that living in socially deprived neighborhoods fosters physical victimization among people with SMI (25, 29). This study further established that residing in rural residences was found to be protective by 52% against sexual victimization, which rhymes with a previous study which revealed that subjective safety for people with SMI is clearly worse in cities than in rural areas (42), while other related studies established that living in socially deprived neighborhoods fosters sexual victimization among people with SMI (25, 29). Based upon results from this study, religion (being Muslim) was protective by 40% against sexual victimization; findings from this study relates to a previous study which suggest that religion is a protective factor against sexual and gender-based violence (SGBV) (43); similarly, a previous study established that trauma related to sexual victimization is shaped by religious beliefs relating to forgiveness, sacrifice and salvation; possibly, people with SMI use religion to cope with the ‘after-effects of sexual abuse’; contrary to findings from this study, a previous study established that manifestation of victimization (including sexual victimization) happens to be more influenced by religion (44).

This study established that participants with a higher socio-economic status (an individual’s position in a society which is determined by wealth, occupation, and social class and is a measure of an individual’s or group’s standing in the community) were found to be less likely to suffer physical victimization compared to those with lower socio-economic status, thus the findings happen to be in agreement with previous studies which suggest that poor social and economic conditions fosters physical victimization among people with SMI (25, 29); most people with SMI happen to be socioeconomically disadvantaged compared to the general population (45), hence more likely to suffer physical victimization than other people without SMI. Similarly, other previous studies have established that socio-economic disadvantage makes persons with severe mental illness to be more vulnerable beyond the effects of the mental illness itself (34, 46).

According to this study, childhood trauma was associated with physical victimization; results from this study are in agreement with previous studies which suggest that increase in the risk of adult physical victimization is associated with previous childhood abuse (47, 48). Similarly, a high prevalence of previous childhood abuse has been previously reported among people with SMI (48–50). It has been suggested that abuse in childhood increases the odds of adulthood violent victimization in both women and men (48). Previous studies established that experiences of childhood maltreatment are associated with more severe psychiatric symptoms and more complex clinical manifestations among people with SMI (48, 51, 52). Possibly, broader stressful childhood experiences may affect the life trajectory negatively in terms of complex social and behavioral outcomes which may increase vulnerability to victimization, rather than there being a specific abusive experiences in childhood that makes people more vulnerable to similar abusive experiences in adulthood (48). Relatedly, experiences of victimization in early life influence risk of later victimization in this causal manner, via changes in social and psychological development and the severity of illness; while on the other hand, the association may simply reflect continuity of adversity across the life course, with early victimization as a marker of social disadvantage that is still present in adulthood, thus increasing risk of victimization (48). Relatedly, this study established that childhood trauma was associated with sexual victimization; findings from this study are in agreement with previous studies which suggest that increase in the risk of adult sexual victimization is associated with history of previous childhood abuse (47, 48). Similarly, other previous studies have reported a high prevalence of previous childhood abuse among people with SMI (48–50, 53). A previous study looking at men and women with SMI found that patients who had been sexually victimized as adults were more likely to have been sexually abused as children, but physical abuse in childhood was not associated with physical victimization in adulthood (39). Relatedly, other previous studies suggest that sexually victimized people with SMI were significantly more likely to report a history of sexual abuse during childhood (4, 39). Among mentally sick people, abuse in childhood increases the odds of adulthood violent victimization (48). Since the cause of sexual victimization is always ultimately the behavior of the perpetrator, it can be difficult to clarify the mechanisms by which a person’s negative childhood experiences increase their vulnerability to later victimization. Grauerholz uses an ecological framework, proposing that personal, interpersonal and sociocultural factors associated with childhood abuse may increase the risk of exposure to potential perpetrators, or increase the likelihood that potential perpetrators will act aggressively (54). Factors associated with childhood abuse in the general population such as lack of resources, social isolation, drug and alcohol abuse, psychiatric symptoms and stigmatization (55–57), may all increase the risk of a perpetrator acting aggressively, due to the perception of the victim as an easier target and feeling more justified in behaving aggressively, as well as decreasing the ability of the victim to respond assertively (54).

According to this study, social support was protective against physical victimization; findings are in agreement with previous studies which suggest that social support happens to be protective against physical victimization among mentally sick people (58); mentally sick people with poorer social support experience greater exposure to traumatic events, while better social support helps ensure a better quality of life for people with mental illness (59). This study further established that social support was found to be protective against sexual victimization; findings are in agreement with a previous study which suggests that good social support lowers the risk of victimization and lessens suffering from exposure to traumatic events (59). Similarly, it has been established that social support networks may serve to buffer the psychological effects of stress and victimization (60).

### Association between physical and sexual victimization with negative outcomes

This study established that risky sexual behaviors (aOR 2.19 95%CI 1.66–2.90; *p* < 0.001) were associated with physical victimization; relatedly, previous studies established that risky sexual behaviors (RSBs) are common among people with SMI (9, 12). Patients in the acute phase of severe mental illness are more likely to practice RSBs, largely associated with the general impairment of reality testing and judgment common among this population (12, 55); since some people with SMI (acutely ill patients) display disturbed behavior which evokes hostile reactions, attempts to control such patients often leads to conflict and physical victimization (23); thus a possible association between RSBs and physical victimization. Risky sexual behaviors were found to be associated with sexual victimization; findings from this study relate to a previous study which suggested a complex link between childhood sexual abuse and adult risky sexual behaviors among persons with SMI (61). Similarly, a previous study established that people with sexual abuse history are significantly more likely to engage in risky sexual behavior than people without sexual abuse history (62). Similarly, a previous research with community-based samples indicated that childhood sexual abuse is associated with increased engagement in risky sexual behaviors during adulthood (63). It has been hypothesized that childhood sexual abuse impacts subsequent risky sexual behavior via three pathways: (1) psychopathology, including PTSD, depression, and dissociation; (2) drug use; and (3) adverse sexual adjustment including an obsession with sexual activities, an inability to sustain intimate relationships, and participation in destructive sexual relationships (64). It has been suggested that for persons with SMI, childhood sexual abuse serves as a threshold for engaging in risky sexual behavior as adults (61). Risky sexual behaviors among patients with a severe mental disorders are highly prevalent and happens to be associated with many negative outcomes (65).

According to this study, mental health stigma was associated with physical victimization; findings rhyme with previous studies which suggest that stigma is associated with prior experience of trauma (59), exceeds the effects of mental illness itself, thus makes people with mental illness to be extremely vulnerable (46); stigma co-occurs with both victimization and serious mental illness (38); obviously, individuals exposed to traumatic events/victimization often feel stigmatized because of their experiences (59). As a possible mechanism, stigma toward vulnerable people can increase the risk of a perpetrator acting aggressively, due to the perception of the victim as an easier target and feeling more justified in behaving aggressively, as well as decreasing the ability of the victim to respond assertively (54).

This study established that mental health stigma was associated with sexual victimization; results from this study rhyme with a previous study which established that sexual victimization among people with SMI is associated with social stigma, shame, guilt, dehumanization and increased vulnerability (66); people with SMI who experience sexual victimization suffer the double burden of stigma from both mental illness and sexual victimization (38, 59, 66). In low-and middle-income countries, lack of psychiatric services and widespread mental illness stigma are structural factors that exacerbate the social vulnerability of persons with SMI (34, 67, 68); stigmatizing attitudes against persons with SMI are widespread in sub-Saharan Africa (40). Relatedly, a previous Ugandan study established that some female participants reported being sexually exploited due to economic and emotional dependence by persons intimate to them (34, 69); economic dependence on intimate partners contributes to Ugandan women’s low negotiating power in decision-making about sex (34, 69). Arguably, the same environmental factors [living in socially deprived neighborhoods, where social and economic conditions foster abusive norms (29)] that are responsible for sexual victimization, trigger/manifest psychiatric symptoms and social stigma since they subject people with SMI to be more vulnerable (25).

Missed taking of oral psychiatric medications in last 3 days was associated with physical victimization (aOR1.50; 95% CI 1.12–2.00; *p* = 0.006), which probably relates to the greater treatment resistance observed among psychiatric patients (70, 71); possibly, administration of treatment against the will of individuals with severe mental illness (23, 72) results into physical victimization. Similarly, missed taking of oral psychiatric medications in last 3 days was associated with sexual victimization, possibly due to the impending relapse associated with non-adherence to antipsychotic medication (73), thus this could have subjected people with SMI to be more vulnerable to sexual victimization.

Poor adherence to oral anti-psychotic medications was associated with physical victimization; findings from this study rhyme with previous studies which established that violent victimization in people with SMI is associated with being unresponsive to treatment and non-adherent with medication (44); greater treatment resistance has been observed among psychiatric patients (70, 71). Relatedly, other previous studies established that lifetime exposure to assault was associated with administration of treatment against the will of individuals with severe mental illness within the mental health care system (23, 72). Poor adherence to oral anti-psychotic medications was associated with sexual victimization; probably, poor adherence to antipsychotic medication increases the risk of relapse and hospitalization and reduces the quality of life (74), thus mentally sick people (with poor adherence to oral anti-psychotic medications) were more vulnerable since they were more likely to relapse and become sexually victimized.

### Strengths and limitations

This study used a large sample size (1,201 participants), established the prevalence of physical and sexual victimization, associated risk factors and their psychosocial consequences among patients with severe mental illness (SMI) in rural and urban Uganda. This study incorporated and determined other very important variables through a variety of standardized tools. In addition, recruiting participants after screening for their insight [recognition of one’s own mental illness and need for treatment; a person’s capacity to understand the nature, significance, and severity of his or her own illness (75)] is a plus strength of the study since inviting and including participants having poor insight might have affected the findings. Limitations of this study are inherent to the cross-sectional study design which did not allow for conclusions about causal pathways but this will be addressed during the longitudinal part of this study. Because of the cross-sectional nature of this study, it is unclear whether potential risk factors were (already) present at the time of physical and sexual victimization; In the future, a prospective design (which is part of the larger study) will be utilized to investigate the risk factors and to capture causal trajectories of physical and sexual victimization among people with SMI in urban and rural Uganda. This study used measures of physical and sexual victimization that were based on self-report, which is unfortunately more apt to be influenced by memory or reporting bias (76); however, recall bias was minimized by obtaining collateral information from significant people to the participants. Similarly, use of self-report was more adequate (especially for this group) than Police reports because people with SMI are less likely to have an official police report about physical and sexual abuse (77). Another limitation was the absence of a neighborhood matched control group. Although results of this study revealed that physical and sexual victimization prevalence is high, it is unknown how much higher these rates are compared with the general population that lives in the same neighborhood or circumstances. To gain more knowledge about the risk factors, it is important to investigate the mediating and moderating factors that influence the risk of physical and sexual victimization. Relatedly, it is important to conduct a study to understand why people with SMI are more prone to physical and sexual victimization, thus more information is needed about lifestyles and related routine activities, type of incidents, the context (e.g., where was it, who was the offender), and the process of cause and effect. Despite the limitations, this study provides additional understanding of the difficult life situation of persons living with SMI in resource-poor settings, documents the physical and sexual victimization by ‘people significant to those with SMI’, community members, Police officers (security personnel), health workers and hospital security guards (especially when people with SMI are admitted in psychiatric settings) during illness episodes. Our findings suggest that it is important for clinicians to assess for physical and sexual victimization among people with SMI in sub-Saharan Africa, in order to provide appropriate support/mental health care.

## Conclusion

People with severe mental illness are victims of physical and sexual abuse. The age group of > = 50 years was more likely to have suffered physical victimization; living in a rural area was protective against physical and sexual victimization; high socioeconomic status (SES) was protective against physical victimization; socioeconomic status (SES) refers to a person’s economic and social position in relation to others, based on income, education, and occupation (78), including possession of commonly available household items/resources (12, 18). Females were more likely to have been sexually victimized; being a Muslim was protective against sexual victimization. Risky sexual behaviors, mental health stigma, missed taking of oral psychiatric medications in the previous 3 days and poor adherence to oral anti-psychotic medications were negative outcomes associated with physical and sexual victimization.

Implication of these findings; practitioners need to consider introducing questions about prior experience of physical and sexual victimization in routine anamnesis which may help uncover physical and sexual abuse among people with SMI to improve upon service delivery. The risk for patients may vary depending on the community in which they live (urban versus rural setting). Physical and sexual victimization among people with SMI is public mental health problem, thus it is important to understand the risk pathways for different types of abuses within a developmental framework. Given the high burden and excess risk of physical and sexual victimization among people with SMI, future research should evaluate complex interventions for improving detection of and response to abusive experiences within mental health services. It is important to conduct studies to guide clinical practice and policy on ‘gender sensitive preventive measures for physical and sexual victimization among people with SMI’.

## Data availability statement

The datasets presented in the study are included in the Supplementary material, further inquiries can be directed to the corresponding author/s.

## Ethics statement

The studies involving human participants were reviewed and approved by the study obtained ethical approvals from the Uganda Virus Research Institute’s Research and Ethics Committee (GC/127/19/10/612) and the Uganda National Council of Science and Technology (HS 2337). The patients/participants provided their written informed consent to participate in this study.

## Author contributions

RM, WS, GR, PA, CB, RR, CT, KG, AK, VP, MN, and EK have made substantial contributions to conception, design, acquisition of data, drafting the manuscript, revising it critically, and gave the final approval of this version to be published. WS did the analysis and interpretation of the data. All authors participated sufficiently in this work and takes public responsibility for appropriate portions of the content.

## Funding

This study was funded by MRC core funding to the Mental health project of MRC/UVRI and LSHTM under the headship of Professor Eugene Kinyanda to undertake the ‘HIV clinical trials preparedness studies among patients with Severe Mental Illness in HIV endemic Uganda (SMILE Study)’.

## Conflict of interest

The authors declare that the research was conducted in the absence of any commercial or financial relationships that could be construed as a potential conflict of interest.

## Publisher’s note

All claims expressed in this article are solely those of the authors and do not necessarily represent those of their affiliated organizations, or those of the publisher, the editors and the reviewers. Any product that may be evaluated in this article, or claim that may be made by its manufacturer, is not guaranteed or endorsed by the publisher.
